# Challenges and Opportunities for Immunoprofiling Using a Spatial High-Plex Technology: The NanoString GeoMx^®^ Digital Spatial Profiler

**DOI:** 10.3389/fonc.2022.890410

**Published:** 2022-06-29

**Authors:** Sharia Hernandez, Rossana Lazcano, Alejandra Serrano, Steven Powell, Larissa Kostousov, Jay Mehta, Khaja Khan, Wei Lu, Luisa M. Solis

**Affiliations:** Department of Translational Molecular Pathology, The University of Texas MD Anderson Cancer Center, Houston, TX, United States

**Keywords:** digital spatial profiling, immune-oncology, biomarkers, tumor microenvironment, pathology

## Abstract

Characterization of the tumor microenvironment through immunoprofiling has become an essential resource for the understanding of the complex immune cell interactions and the assessment of biomarkers for prognosis and prediction of immunotherapy response; however, these studies are often limited by tissue heterogeneity and sample size. The nanoString GeoMx^®^ Digital Spatial Profiler (DSP) is a platform that allows high-plex profiling at the protein and RNA level, providing spatial and temporal assessment of tumors in frozen or formalin-fixed paraffin-embedded limited tissue sample. Recently, high-impact studies have shown the feasibility of using this technology to identify biomarkers in different settings, including predictive biomarkers for immunotherapy in different tumor types. These studies showed that compared to other multiplex and high-plex platforms, the DSP can interrogate a higher number of biomarkers with higher throughput; however, it does not provide single-cell resolution, including co-expression of biomarker or spatial information at the single-cell level. In this review, we will describe the technical overview of the platform, present current evidence of the advantages and limitations of the applications of this technology, and provide important considerations for the experimental design for translational immune-oncology research using this tissue-based high-plex profiling approach.

## Introduction

In the 21st century, surgery, chemotherapy, and radiation therapy remain the leading cancer treatment options for patients around the globe. However, these “traditional” therapies target mainly tumoral cells, and currently, it is well known that cancer initiation and progression also involve the tumor microenvironment (TME) (1). Recently, the development of emerging targeted therapy for precision oncology, especially in the domains of immunotherapy, requires the identification of biomarkers in tumor and cells of the TME that can predict therapy efficacy and of signaling pathways that can help to understand tumor biological behavior (2, 3).

The TME is a dynamic entity composed of malignant cells and the surrounding non-malignant cells and tissue structures; these include immune cells, stromal cells, nerve fibers, blood vessels, and the extracellular matrix. It has been shown that tumor cells acquire capabilities to overcome the normal tissue anti-tumoral homeostasis through interactions among malignant cells and surrounding non-malignant cells, creating a favorable environment to grow, invade, and metastasize (4–6). Thus, studying the immune profile of tumors has become an essential tool for the development of new cancer therapeutic strategies such as immunotherapy (2, 3).

Immunoprofiling consists of measuring and characterizing the immune system to acquire information on how immune cells can respond to different diseases and therapies (7). Immunoprofiling in tumor tissue may represent a challenge when small tissue samples and tissue heterogeneity limit the assessment of biomarkers; therefore, strict selection of samples and a sufficient high-quality material assessed by a pathology quality control is needed (8, 9). To overcome tissue limitation, multiplexed and high-plex technologies have become essential tools in immunoprofiling research, allowing the simultaneous identification of multiple specific proteins or molecular expressions in restricted tissue sample (10). Tissue-based assays for immunoprofiling allow the visualization of pathological and phenotypic features and the identification of different levels of histological and biomarker heterogeneity that can provide important clues in tumor biology and biomarker discovery and therefore should be carefully considered for the experimental design of translational oncology research studies (11, 12).

The GeoMx Digital Spatial Profiler (DSP) is a novel high-plex protein and RNA platform especially useful in limited tissue samples. It quantifies the abundance of protein or RNA by counting unique indexing oligos assigned to each target of interest. The use of oligonucleotides allows the study of a higher number of biomarkers compared with other techniques such as multiplex immunofluorescence (mIF) or co-detection by indexing (CODEX), but limits the study of single-cell expression and spatial analysis. DSP is also a non-destructive technique, and slides can be used for other studies after the assay is completed. This technology has been attractive to many investigators because of its potential to provide deep insight into tumor immunology, tissue heterogeneity, and biomarker discovery (13).

In this review, we will provide a brief technical overview of the platform, we will describe current evidence of the advantages and limitations of the applications of this technology in translational immune-oncology research, and we will list several considerations for the experimental design of immune-oncology translational research studies,

## Technical Overview of the DSP Platform

**Figure 1:**
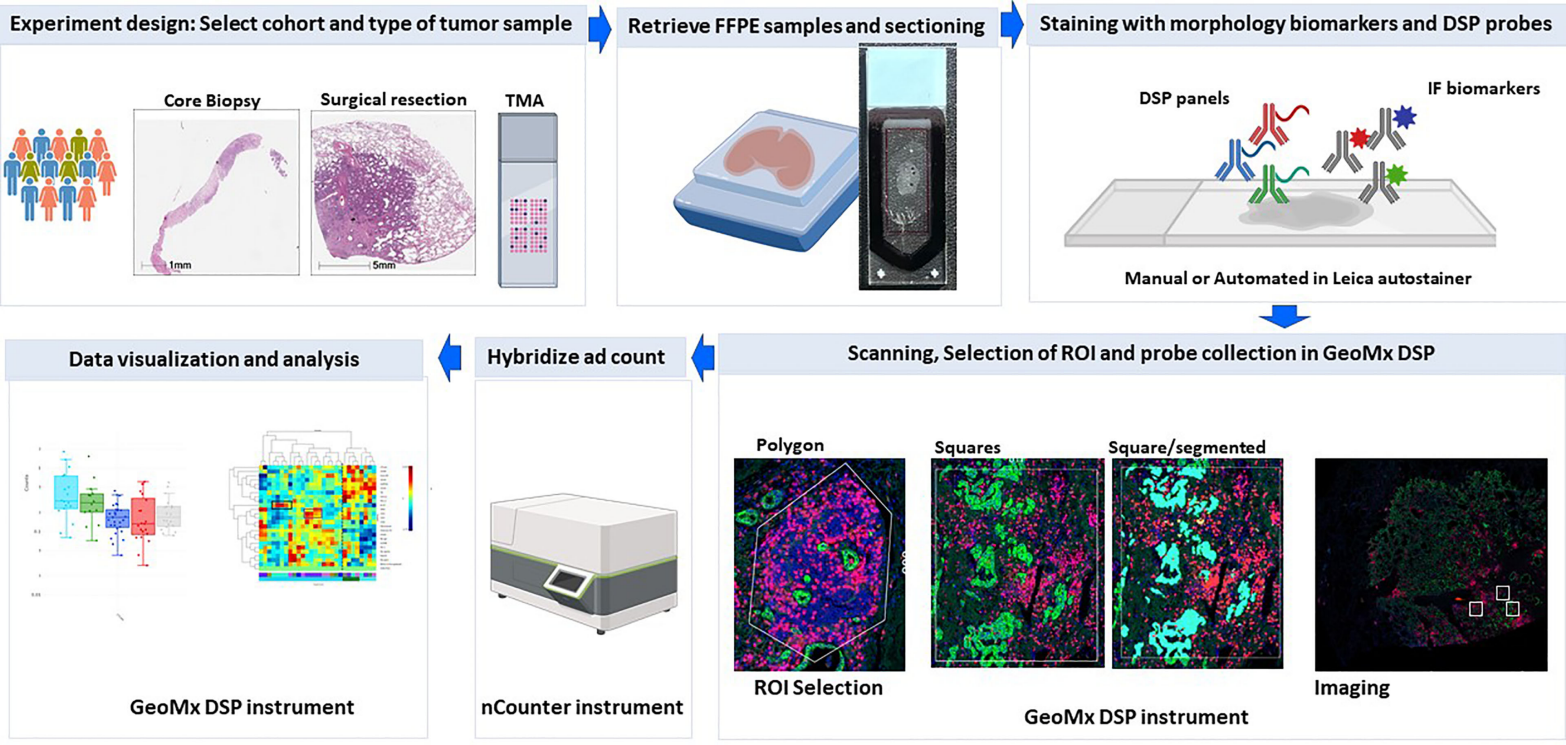
DSP assay workflow. Schematic picture showing the workflow of profiling using the Geomx DSP.

The nanoString GeoMx DSP is a tissue-based assay for high-plex profiling of protein and RNA within specific areas of interest. The main steps included in the workflow of this platform include tissue preparation with immunofluorescence biomarkers and DSP probes, regions of interest selection, oligo collection, hybridization, and counting and data analysis (**Figure 1**).

### Tissue Preparation

This technique is compatible with formalin-fixed paraffin-embedded (FFPE) and frozen tissue. For both types of samples, sections of 5-μm thickness should be obtained following the specifications of sample preparation guidelines from nanoString (14). Then, deparaffinization and antigen retrieval are performed similar to the standard immunohistochemistry assay using manual or an automated stainer platform. Then, a single step of reagents is applied to the tissue, which consists of a cocktail of immunofluorescence biomarkers and probes or antibodies linked to photo-cleavable DNA tags. The immunofluorescence biomarkers will be used as visualization markers (VMs), and they include a DNA marker (SYTO13) and up to three specific antibodies or RNA probes conjugated to fluorophores (13, 15).

### ROI Selection

Once the incubation is complete, slides are loaded onto the DSP instrument, and scanned to produce a digital image that displays the tissue with histological features highlighted by the fluorescent VM. Then, ROIs of different sizes (up to 660 * 785 µm) and shapes (rectangles, squares, and free hand shaped polygons) can be selected. These ROIs can be placed in different areas defined by biomarker expression, spatial location, and/or morphological features. For example, the ROI can be selected in immune-depleted areas or immune-enriched areas based on CD45 expression in the tissue sample; in tumor invasive margin vs. central tumor areas based on spatial tumor location; or tumor tissue vs. normal-appearing tissue based on the morphological characteristics of the tissue sample. After ROI selection, at the discretion of the investigator, these regions can be segmented in more than one compartment using the VM and assisted by an image analysis software embedded in the DSP device; the compartments can be defined as malignant epithelial cells vs. stroma (based on pancytokeratin expression), or in individual sets of cell populations such as CD45+, CD3+, or CD68+ cells (10, 11). These morphology biomarkers and histologically driven region of interest selection allow an integrated analysis of the protein or RNA expression with pathological features and within the context of diverse elements of tissue samples.

### Oligo Collection

After the ROIs are selected, and compartments have been segmented, these areas are exposed to ultraviolet (UV) light using a programmable digital micromirror device (DMD). This UV light process explains the term “Area of illumination” (AOI), which is used to refer to an entire ROI or compartment that will be illuminated in order to cleave DNA tags in a region-specific manner. The DMD will autoconfigure to match the exact spatial pattern defined previously in each ROI of each tissue section. The released indexing oligos are collected *via* microcapillary aspiration, and dispensed into a microplate (16).

### Hybridization and Counting

After collection of indexing oligos has been performed, they are hybridized to optical fluorescent barcodes or GeoMx Hyb codes. Then, they are digitally counted using the single-molecule counting nCounter System or analyzed using next generation according to manufacturer’s instructions (NanoString, Seattle WA) (16, 17).

### Data Output and Analysis

The counts obtained are then mapped to the different areas selected in the GeoMx DSP device; the device has an analysis suite that facilitates quality control (QC) of DSP counts, and data visualization, normalization, and analysis. The QC of the initial dataset is the initial step for data analysis and comprises the assessment of parameters such as binding density, positive and limit of detection controls, positive control normalization, and minimum nuclei and surface area. The normalization step allows the normalization of the data using the counts from specific probes like “Housekeepers” or “IgGs” for background correction. The method of normalization should be decided based on the type of sample and the aim of the project. Data can be visualized in different ways including heatmaps, boxplots, and correlation plots. The platform also provides statistical test functions (18).

## Profiling of Different Types of Tumors Using DSP

Currently, histological and cell phenotype biomarker features are important considerations to develop strategies for immune profiling in tumor tissue, including profiling using DSP. Tumors display several levels of histological, immune, and molecular heterogeneity, such as diverse morphological features of tumor cells; different architectural tumor arrangements, quantities, and spatial infiltration of cells of the tumor immune microenvironment; composition, distribution, and density of the extracellular matrix; or genomic, transcriptomic, and proteomic heterogeneity (19–21). This heterogeneity is observed not only among individuals, but also in different areas from the same tumor; moreover, heterogeneity can be dynamic and temporal, and tumor features change at different stages of the diseases, at different time points of treatment, or even influenced by the nervous system or by the diversity of the patient gut microbiome (22–24).

Strategies for immunoprofiling with DSP must consider several histological features that are visible using standard microscopy or highlighted by the use of immunofluorescence biomarkers. For example, similar to other multiplex approaches, to profile specific regions of interest (ROI), the experimental design must utilize specific biomarkers for tumor identification, which will be different in tumors that originate from epithelial cells such as carcinomas compared to tumors that derive from other cells of origin such as gliomas, lymphomas, or sarcomas, which not only have a different phenotypic profile but also have different architectural histological arrangements (25–27). Similarly, when profiling immune cells, the strategies for selection of ROI may be different even among tumors that share a similar histological type, and the immune microenvironment may vary from tumors that are immune “cold” to highly inflamed “hot” tumors with the presence of immune aggregates organized in tertiary lymphoid structures (TLSs). In the same lines, the immune infiltrate in tumors can also be heterogeneously distributed, and some tumors present immune infiltration only in the periphery while the central tumor area is immunologically “cold”, constituting the immune excluded tumor type; thus, strategies should take these features into consideration (28, 29). Another example of heterogeneity that must be taken into account is the temporal heterogeneity of the different expression of biomarkers like PD-L1 in different stages of tumor progression, or the changes of histological type and mutation status during lung cancer targeted therapy (30, 31). Thus, monitoring the changes on biomarker profile within the context of histological features is extremely important for immune-oncology research.

In recent years, several investigators have utilized the DSP to profile several types of tumors, using different morphology biomarkers tailored to the investigator’s research question and with different strategies for regions of interest selection and segmentation. In this section, we will highlight selected peer-reviewed publications that have used this platform, and show the feasibility of its implementation as a tool for translational oncology research, with emphasis on the advantages and limitations found among investigators in the research community.

### Carcinomas

The DSP platform in carcinomas has been used mainly to study the differential expression of biomarkers at the tumor and stroma compartments identified by the expression of cytokeratin and to compare different cohorts defined by clinicopathological characteristics or specific biomarker expression of tumor samples. It is worth noting that carcinomas are a heterogeneous group of malignant neoplasms of epithelial cell origin (32), which can be classified according to their histologic subtype (squamous cell carcinoma vs. adenocarcinoma) (33) or according to tissue of origin (e.g., lung or breast carcinomas) (34). Even when arising from the same organ, carcinomas may have a wide variety of growth and differentiation patterns, depending also on their histological subtypes; as an example, basal cell carcinoma variants can have a nodular, superficial, micronodular, infiltrative, or metatypical pattern (35) and lung adenocarcinoma may have solid, papillary, or acinar growth patterns (36). Since epithelial cells contain keratins as intermediate filaments and keratin antibodies are widely used to identify tumors of epithelial origin (37) being expressed in up to 93% of carcinomas (38), an antibody cocktail that recognize acidic and basic cytokeratins [pancytokeratin (panCK)] is the standard biomarker to highlight epithelial cells in mIF approaches including DSP. Nevertheless, some carcinomas may express very low levels of panCK or completely lose their expression in poorly differentiated or metastatic samples (39). Carcinoma cells are supported by tissue stroma composed of carcinoma-associated fibroblasts, endothelial cells, and the extracellular matrix. Stromal interactions with malignant cells promote cancer growth and invasion (40, 41). These stromal interactions also include the characterization of tumor-infiltrating lymphocytes (TILs). To achieve a comprehensive TIL characterization, many guidelines have been published to standardize the definition of the stromal area and its relationship with immune cell infiltrates, especially in the case of breast ductal carcinomas (42, 43). Therefore, biomarkers that aid in the identification of immune cells or immune cell subsets have been used as VM using DSP.

### Breast Carcinomas

Different studies have used DSP to profile breast cancer samples. As in most of carcinomas, many of these studies rely in panCK as a VM to highlight epithelial tumor cells. Some of them segmented the ROIs in tumor and stroma based on the expression of panCK (44, 45) while others collected probes from non-segmented ROIs (46, 47). The performance of the DSP was assessed in this tumor type by assessing levels of protein or RNA signal and associating the DSP counts of different biomarkers to traditional IHC scores for DSP protein panels or bulk RNA data for DSP RNA panels; overall, the results show a robust protein signal and moderate to strong associations between protein DSP counts and IHC and RNA DSP counts with Bulk RNA data for the majority of the targets (45). The utility of this platform has been shown in a wide spectrum of FFPE samples from whole tissue surgical resections, core biopsies, and tumors placed in tissue microarrays (TMAs) (48). For example, this platform allowed the identification of differential protein expression of immune markers in tumor vs. stromal compartments of early-stage triple-negative breast carcinomas (TNBCs) categorized based on the expression of PD-L1 companion assays, and the findings showed potential application in the development of more effective immunotherapies and associated biomarkers of response (44); other studies have also used the platform in TNBC to quantitate HLA-DR proteins and immune biomarkers and find potential prognostic biomarkers (47), and to find differential protein signature expression associated to the outcome of TNBC patients treated with chemotherapy, revealing the utility of the platform to identify biomarkers associated with response and resistance to adjuvant treatment (49). Furthermore, spatial profiling assessed in breast tumor samples treated with Her-2-targeted therapy allowed the identification of biomarkers that can predict tumors’ pathological complete response, and the assessment included intra-tumor heterogeneity and biomarker changes at different time points of therapy (46). Among the limitations found on these analyses, it was noted that the lack of integration of data from other assays limited the development of complex signatures related to prognostic or subtypes of breast cancer, and that the inability of H&E image data or capabilities to generate a pseudo-H&E projection from immunofluorescence-stained slides limited the morphological evaluation of these samples. In addition, it was noted that although single-cell profiling is not available for protein expression, deconvolution may be achieved with RNA analysis of large panels such as cancer transcriptome atlas or whole transcriptome atlas (48). Of note, a recommendation of best practices for DSP profiling was published with the purpose to standardize and promote the collection and analysis of high-quality data (48).

### Non-Small Cell Lung Cancer

The DSP platform has been used in a variety of NSCLC studies. Most of these studies have employed panCK to define the tumor compartment, along with immune markers such as CD45, CD3, CD8, and CD68 to define different immune compartments for ROI segmentation (50–52), Among the advantages of DSP profiling in these tumor types was the possibility to interrogate a high number of biomarkers into different spatial types provided by the VM, such as the expression of PD-L1 in tumor cells (defined by panCK-positive expression) or macrophages (defined by CD68 expression). Similar to breast cancer, expression of biomarkers found in tumor vs. immune or stroma compartment was distinct. The use of this platform in lung cancer research was found to be attractive to discover biomarkers that can predict response to immunotherapy in NSCLC, and to allow a more comprehensive understanding of the immunological parameters that influence patient outcome (50, 52). Among the limitations in the application of this technology, it was noted that the size of ROI is limited to a maximum of 600-µm geometric shapes, which may be inconvenient as it could not cover a wider area of interest, such as the need to improve the workflow to determine the method of normalization and proper identification of probes that lack robust signal-to-noise ratio (SNR). In these studies, it was noted that some markers have poor signal-to-background ratios such as PD-L1, which should be expected to be higher in a subset of NSCLC tissues, as well as ARG1 in the immune compartment (50, 51). These data highlighted the need for more rigorous validation of the antibodies used in the DSP panels and the importance of validation of the data obtained using orthogonal approaches. Of interest, one study evaluated the dynamic range and reproducibility of PD-L1 expression obtained by DSP protein panel assay using a TMA with isogenic cell lines expressing various levels of PD-L1. The data showed a dynamic range of SNR and DSP counts according to the levels of PD-L1 expression expected in the isogenic cell lines, high correlation of PD-L1 digital DSP counts with immunohistochemistry scored with quantitative software and with quantitative fluorescence, high reproducibility between runs, and consistency of the analysis in slides with short and longer slide storage. Although this study did not include assessment using tumor tissue, the data suggested potential clinical applications of this technology (17).

### Other Carcinoma Types

The DSP platform was also used in other carcinoma types, such as head and neck squamous cell carcinoma (HNSCC), prostate adenocarcinoma, colorectal carcinoma, and cholangiocarcinoma. In HNSCC, DSP was used in one study employing protein panels and panCK, CD3, and CD8 as VM. Kulasinghe et al. aimed to determine the protein expression of immune biomarkers in a cohort of patients receiving immune checkpoint therapy reported as non-progressive vs. progressive disease and to identify biomarkers predictive of therapy; in addition, the study also compared these results with mIF staining, which included a limited biomarker panel with panCK, CD8, PD-L1, and DAPI. The DSP platform allowed the determination of markers involved in the beneficial effects of immunotherapy with a greater depth of multi-plexing beyond conventional IHC; these data also showed reproducibility and high concordance between DSP counts, with the results of mIF suggesting the robustness of the assay (53).

In prostate cancer, the utility and performance of DSP were determined in one study. Brady et al. used DSP technology to quantitate protein and RNA abundance in spatially distinct regions of metastatic prostate cancer (mPC) samples placed in a TMA. The experimental design included different samples from the same patient, and some of these samples were decalcified bone metastasis samples. The VMs used were panCK, CD3, CD45, and SYTO13 that aid in the selection of no segmented circular ROI (500 microns diameter) per core based on the highest percentage of tumor cells; each ROI was also assessed by tumor cell composition. The investigators employed 2 DSP panels in serial sections, a DSP protein panel with 60 oligo-conjugated antibodies with the addition of androgen receptor (AR) and synaptophysin (SYP), and a 2,093-gene RNA panel. Using this platform, the investigators were able to define tumor phenotype, measuring tumor heterogeneity by assessing the spatial composition of metastases, and they found a common ground in the phenotype classification between metastases from the same individual and the highly consistent expression of proteins across the multiple ROIs within each tumor, indicating low intra-tumoral heterogeneity and detecting features of tumor biology that are currently associated with specific therapeutics. In addition, they found a general lack of immune cell infiltrates in the vast majority of metastases and a high expression of the immune checkpoint proteins. Of note, in this study, FFPE archived samples showed no age-related variation and bone metastasis samples had lower probe counts compared to soft tissue cores; however, high concordance between bone metastasis and soft tissue was found. It is worth mentioning that when they compared the DSP counts vs. bulk tumor RNAseq, although most of the biomarkers have high concordance, there were some discordant cases, which may limit the capability of DSP to detect splice variants, when compared to traditional approaches (54).

The DSP platforms were also used to validate scRNA-seq data and to investigate spatial defined gene expression in a small cohort of CRC tumors. Pelka et al. performed DSP RNA analysis using tissue sections from 3 tumors that showed high CXCL13 T-cell activity obtained by scRNA-seq data, and the investigators measured ∼1,500 genes using 45 circular ROIs measuring 500 μm in diameter placed in tumor areas; these ROIs were segmented into panCK-positive (epithelial) and panCK-negative (non-epithelial) segments. Their findings demonstrated a correlation between the CXCL13 signal in panCK-negative regions and the interferon-stimulated genes/MHC-II signal score in panCK-positive regions, suggesting potential interactions between malignant cells and T cells in this subset of samples (55).

In cholangiocarcinoma, Aguado-Fraile et al. studied the molecular and morphological effects of mutant IDH1 inhibitor ivosidenib (AG-120) in mutant IDH1 cholangiocarcinomas in seven paired pre- and post-treatment FFPE samples. Samples were stained with panCK, SYTO13, and CD45 immune cells to select 12 ROIs, 6 in tumor-rich areas and 6 in tumor-rich immune-infiltrated areas and incubated with a protein panel. DSP was capable of showing reduced levels of expression of different markers such as panCK, EpCAM, and CK19 in tumor cells of post-therapy biopsies when compared to pre-treatment biopsies, suggesting a switch in differentiation program, which was also suggested by the analysis of morphological features. AKT phosphorylation levels were decreased upon treatment with ivosidenib across the samples, and PD-L1, PD1, and VISTA/B7-H5 were increased in post-treated tumor-infiltrated immune cells. These findings support the rationale of combining mIDH1 inhibition with checkpoint inhibition in patients with mIDH1 cholangiocarcinoma (56).

### Melanomas

Malignant melanoma is a neoplasm produced from melanocytes that undergo malignant transformation (57). Melanomas are visualized in FFPE tissue slides with antibodies such as S100B (58), PMEL17, and HMB45 (anti-PMEL17/gp100) (59, 60). Digital spatial approaches have been widely used in this tumor type with publications showing the feasibility of this platform for biomarker discovery.

Vathiotis et al. used a combined modality of bulk mRNA and spatial DSP protein model to obtain a more detailed biological information of data related to immune regulation and other aspects of the tumor–stroma interaction in immunotherapy-treated melanoma samples. To accomplish spatial analysis, they used a TMA stained with a panel of 44 proteins and S100/HMB45, CD45, and CD68 as VMs to segment melanocytes (S100/HMB45+), tumor-infiltrating lymphocytes (CD45+), and tumor-infiltrating macrophages (CD68+). For mRNA, they performed bulk mRNA gene expression. Positive correlation of bulk mRNA and protein DSP counts was observed for most targets and was higher when comparing bulk mRNA and proteins from the tumor compartment; of note, some targets did not correlate, or correlated inversely. The combination of platforms proved to be superior in predicting response to ICI and clinical outcomes than either of the platforms alone (61).

Toki et al. performed digital spatial profiling using a 44-plex antibody cocktail to search for protein expression that could potentially be used to predict response to immune therapy in melanoma, using a cocktail of antibodies against S100 and HMB45, CD68, and CD45, to identify and segment ROI in tumor cells (S100/HMB45+), macrophages (CD68+), and leukocytes (CD45+) (62). In addition, concordance with annotated data obtained by automated quantitative fluorescence (AQUA) was assessed with this technology, and authors found the correlation of several biomarkers with prolonged overall survival and prolonged progression-free survival, as well as response to immunotherapy; the investigators also found high concordance between DSP counts and the data obtained from AQUA. Among the main limitations of the DSP assay was the limited resolution, which resulted in missing cells for profiling and misplacement of cells to a different compartment (62).

In another study, Cabrita et al. used clinical samples of metastatic melanomas to investigate the role of B cells in antitumor responses. They used a DSP protein panel of 60 immune-related proteins in samples placed in TMAs. Antibodies against CD3, CD20, and HMB45 plus S100B were used for VM and used to segment the ROI in B cells (CD20), T cells (CD3), and melanoma cells (PMEL/S100B), and the tumors were categorized into tumors with and without TLS. This analysis showed that T cells from tumors without TLS have a dysfunctional phenotype, thus indicating that TLS has a key role in sustaining an immune-responsive microenvironment (63). In a similar study, Helmink et al. also used DSP to profile melanoma samples with a high-risk resectable disease treated with neoadjuvant immunotherapy. The VMs were CD3, CD20, and HMB45, and then ROIs were placed in tumor areas associated and not associated with TLS and segmented in T-cell compartments based on CD3 expression. They found a higher expression of biomarkers of T-cell activation in TLS-associated T cells (64).

### Gliomas

Gliomas are intra-axial tumors that originate from glial cells. They can be classified based on different degrees of differentiation, with glioblastoma multiforme (GBM) being the most common grade IV glioma subtype (65). GBMs can be further classified into genetic alteration subtypes, such as IDH-wild type and IDH-mutant type (66). GBMs are very heterogeneous tumors with several histopathological patterns (67). The most common glioma biomarker is the glial fibrillary acidic protein (GFAP), which is expressed by normal glial cells and malignant glial cells (65).

GFAP and CD3 have been employed by Barber et al. as VMs using DSP in 10 cases of MGMT methylated versus unmethylated IDH-wild-type glioblastoma. The objective of the study was to assess 31 immuno-oncological protein targets. Six ROIs per section were selected by aligning fluorescent images with H&E images with ROIs predetermined by a certified neuropathologist. The investigators’ main finding was the identification of immune biomarkers differentially expressed in MGMT methylated tumors compared to unmethylated GBM. The main limitations of this study were the absence of single-cell expression data, the lack of information of molecular pathways activated in the regions selected, the complex normalization process, and technical aspects that can lead to operator-dependent errors and variability (68).

### Hematological Malignancies

There are very limited studies on hematological malignancies and DSP assay. One study of Koldej et al. examined the feasibility of applying DSP assay to analyze archival diagnostics of bone marrow (BM) trephine samples fixed in B5 and decalcified in an acid solution. The investigators used a DSP protein panel and CD3 and CD45 as VM and selected non-segmented ROI based on dual CD3 and CD45 staining. This study has shown that these archival samples can be used for analysis of the BM microenvironment. The investigators also noted challenges with housekeeping normalization and nuclei count was used as a normalization method; this was probably due to the specific histological and biological features of this type of samples. In addition, another major limitation of the DSP technique in these samples was the inability to obtain single-cell resolution (69). Vadakekolathu et al. also employed a DSP protein panel in BM biopsies from patients with acute myeloid leukemia; the investigators profiled T cell-rich and myeloid cell-rich ROIs based on CD3 and CD123 VM, and annotated the ROIs based on enrichment of T cells (CD3). They found differential expression of protein immune biomarkers between T cell-infiltrated and T cell-depleted AML subtypes and between T cell-rich and T cell-poor ROIs, indicating biomarker heterogeneity. Notably, they also identified protein signatures associated to outcome in pre-treatment samples (70).

In summary, current publications highlight the feasibility to apply this technology in different tumor tissue samples with the purpose of studying differential expression of biomarkers “spatially” defined by histological and biomarker compartments to answer several important questions in translational oncology research. Multiple studies have shown that the data obtained with this technique have a high concordance compared to the data obtained by other profiling technologies such as single IHC, multiplex IF, and bulk RNA analysis for most of the biomarkers, but not for all. The main limitations of this platform are the inability to provide single-cell resolution of the DSP panels, thus limiting the understanding of cell–cell interaction, and the different approaches for normalization and data analysis. These studies also highlighted several steps on the workflow of this platform that will result in variability that will eventually have an impact on the interpretation of the data. Several considerations for the experimental design based on our own experience and highlighted in the above peer-reviewed studies are described in the next section.

## Experimental Design Considerations for Immune-Oncology Translational Research Using DSP

Similar to other tissue-based profiling techniques, the experimental design should be tailored to specific research questions and follow standardized protocols that consider the tissue selection, analytical validation of morphology biomarkers, harmonized strategy of ROI selection with full annotation of histological and biomarker features, several steps in quality control from tissue-related steps to data interpretation, and a standardized data management workflow. In this section, we will describe the steps developed and followed in our laboratory for our different assays.

### Tissue Selection and Pre-Analytic Pathology Quality Control

Tissue selection is mostly based on quality and quantity. It is recommended to select tissue that has been processed adequately to avoid pre-analytical variables. For RNA analysis, samples should be fixed in 10% neutral-buffered formalin for more than 16 h, and their FFPE blocks should not be more than 4 years old (71). The GeoMx guidelines recommend tissue fixation in 10% neutral-buffered formalin for 18 to 24 h at room temperature; this applies to tissues less than 0.5 cm in thickness (72).

Although the DSP is a useful platform for small samples and limited tissue, it is best to avoid samples with large areas of necrosis, hemorrhage, and artifacts. Also, due to the low SNR obtained from some targets, it is not recommended to select ROIs containing less than 20 cells for protein assays and less than 200 cells for RNA assays (73); thus, we must secure these cell counts in at least one area of our tissue section. Furthermore, for normalization purposes, it is best to select similar number of cells among different AOIs, always considering the type of tissue sample that will be used for the assay.

There are several types of tissues that may be available for translational research, including the following:

*a) Whole tissue sections from surgically resected tumors:* This type of tissue sample may allow for the identification of ROIs in a wide range of areas with different histopathological characteristics. In the context of immunoprofiling, these can include areas with different degrees of immune infiltration by CD45+ cells, different tumor cell quantities, different patterns of tumor growth, and different histological architectural features such as intra-tumoral, invasive margin, or peritumoral areas (74).
*b) Whole tissue sections from core needle biopsies*: Tissue samples of this type are usually of limited size and therefore restrict the selection of ROIs to the width and length of the core biopsy, which are usually between 0.8 and 1.5 mm wide and no more than 20 mm long, depending on the type of tissue and the needle gauge used by the clinician (75). Important factors, such as an inadequately low number of tumor cells and the presence and level of preservation of architectural features of the tumor stroma, must be followed to determine the eligibility of the sample for protein profiling. For example, disaggregated tumor tissue, or tumor tissue with extensive hemorrhage or immersed in extensive necrosis, may not be adequate for analysis.
c) *Sections obtained from TMAs:* TMAs are produced by extracting cylindrical tissue cores from different paraffin donor blocks and re-embedding these specimens into a single recipient (microarray) block at defined array coordinates. Using this technique, several tissue samples can be arrayed into a single paraffin block and analyzed for biomarker research at a reduced cost (76). TMA cores can be selected in a range from 0.6 to 6 mm. Since the area that can be scanned by the DSP platform and the ROIs used to define AOI have certain limitations (DSP scan area, 36.6 × 14.6 mm) (AOI maximum size: 660 × 785 μm), the TMA needs to be constructed in a block that can be covered by the DSP scan area and the sections need to be positioned in the center of the slide. Of note, the AOI will not cover the entirety of a 1-mm-wide TMA core, and some cores will not have enough tissue to place AOI (72).
*d) Cytology specimens:* This type of specimen includes fine needle aspiration (cell blocks), which is alcohol-fixated, and smears, which are air dried. Currently, however, the DSP assay has not been validated for this type of sample, and therefore, similar to IHC, the best laboratory practice is to perform a separate validation that includes this type of sample. Of note, tissue architecture may be lost and a scarcity of cells may represent a challenge when selecting AOIs with adequate numbers of cells.
*e) Decalcified specimens:* Decalcification procedures affect antigenicity at different levels, depending on the decalcifying agents used. Publications that use decalcified tissue, such as BM biopsies, for GeoMx DSP assays and obtain optimal results are available, but the laboratory should test enough tissues to ensure that the assay consistently achieves the expected results especially when assessing RNA expression (69, 77). After the samples have been selected, sectioning and processing must follow the manufacturer instructions.

After the tissue is incubated with DSP probes and VM, the tissue slides are scanned in the DSP platform to collect DNA tags. In our experience, and considering all the steps of the process, our laboratory is able to run up to 8 slides per week, and scan 4 slides per day for one DSP device. However, this also depends on the quantity of ROIs, which may prolong the processing of the samples during the hybridization step. Several considerations must be taken to select the slides for each run depending on the scientific question, and to avoid batch effects, it is recommended to randomize samples from different cohorts, for example, mix samples from patients who have received therapy with samples from untreated patients, recurrent and non-recurrent tumors, primaries and metastasis, etc. Ideally, this randomization must be performed with the aid of a data analyst, a statistician, or computational bioinformatics. The laboratory also includes an external control at different runs to evaluate reproducibility among runs.

### VM Validation and Tissue Preparation

**Figure 2:**
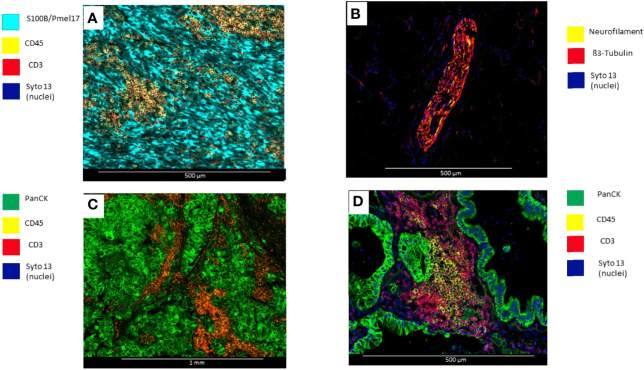
Microphotographs showing immunofluorescence biomarkers used to visualize different elements of tumor tissues. **(A)** Surgical resected uveal melanoma sample stained with S100B/Pmell7 (melanoma cells), CD45 (immune cells), and CD3 (T-cells). **(B)** Surgical resected colon sample with a peripheral nerve identified by β3-Tubulin and Neurofilament. **(C)** Surgical resected breast carcinoma stained with PanCK (tumor), CD45 (Immune cells), and CD3 (T-cells) **(D)** Surgical resected lung carcinoma stained with PanCK (adenocarcinoma cells), CD20 (B-cells) and CD3 (T-cells).

*a) Selection of morphology biomarkers:* With fluorescent antibodies, up to four morphology biomarkers can be used to visualize tissue components and guide the selection of ROIs. Excitation with UV cleaves the DSP barcodes from their probes or antibodies. Of note, DAPI staining is not compatible with DSP (**Figure 2**) (78).
*b) Optimization of custom morphology biomarkers:* Depending on the scientific question, other morphology biomarkers for immunofluorescence that are not included in the commercially available NanoString morphology kit may be needed and can be incorporated after rigorous validation to achieve a high SNR and specificity. Antibodies should be optimized first with standard IHC, by using adequate controls, tissue, or cell lines with different levels of expression of the protein of interest and following analytical validation recommendation for IHC assays (79–83). After this, we perform an antibody optimization with immunofluorescence where a conjugated antibody is tested in a control tissue in isolation and then as a cocktail with SYTO13 and other fluorophores. The IF biomarkers used for DSP need to be conjugated with specific fluorophores (AF532, AF594, and AF647). The first step is to test antibodies that are already conjugated with fluorophores compatible with the DSP assay. Alternatively, carrier-free antibodies can be conjugated with the relevant labeling kit.

### Strategies for ROI Selection and Compartment Segmentation

Once the slides have been processed for DSP assay and scanned in the DSP device, there are certain pathology quality assurance measures that must be performed to ensure that the technique has worked properly.

*a) Verification of IF staining quality of morphology biomarkers:* The pathologist evaluates if the stained morphology biomarkers perform as expected. This step is also important to verify that the morphology biomarkers employed in the assay fit the specific strategic design and is especially important if the laboratory uses several morphology kits and biomarker combinations.
b) ROI selection strategy:

#### Tissue Location

ROIs can be selected based on histological architectural patterns in tumor tissues or by the expression of any specific morphology biomarkers. NanoString’s commercially available morphology biomarker kit for solid tumors and melanoma allows the user to select the ROI based on the following features:

### Levels of Immune Infiltration

Areas that have high or low immune cell infiltration can be identified with staining against CD45.

### Different Histological Features or Tumor Spatial Organization

These areas can be identified using tumor biomarkers (CK, S100B/Pmel17, or S100/HMB45) for the purpose of selecting ROIs based on the histological architecture of a sample (tumoral vs. non-tumoral areas, *in situ* vs. invasive tumor, invasive margin vs. central tumor areas).

### Types of ROIs

**Figure 3:**
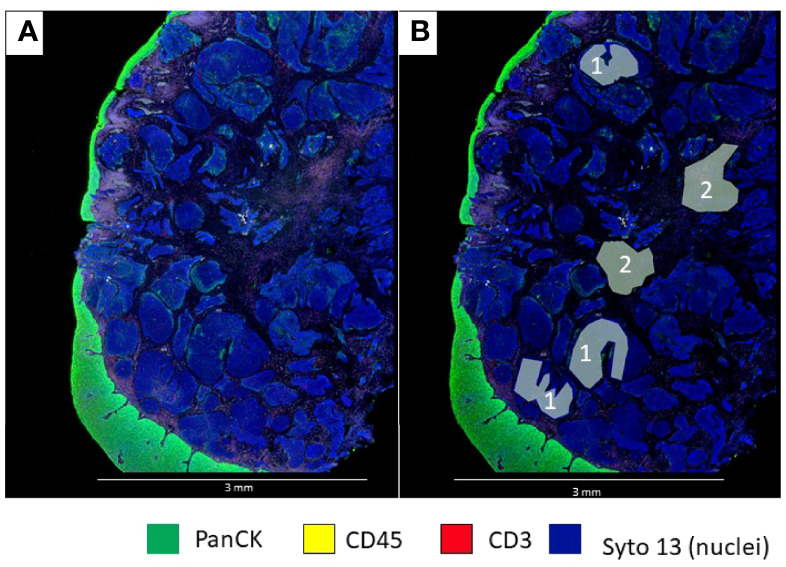
Microphotographs showing a section of a biopsy with invasive basaloid rectal carcinoma tissue. **(A)**, Multiplex immunofluorescence performed with DSP assay highlight epithelial cells with PanCK (green) and nuclei with Sytol3(Blue), Carcinoma nests showed low levels of panCK expression while superficial squamous epithelium shows strong panCK expression, **(B)** Marked up image showing ROI selection in tumor cells and tumor stroma, ROI strategy to identify carcinoma from tumor stroma is only possible with non-segmented polygons.

**Figure 4:**
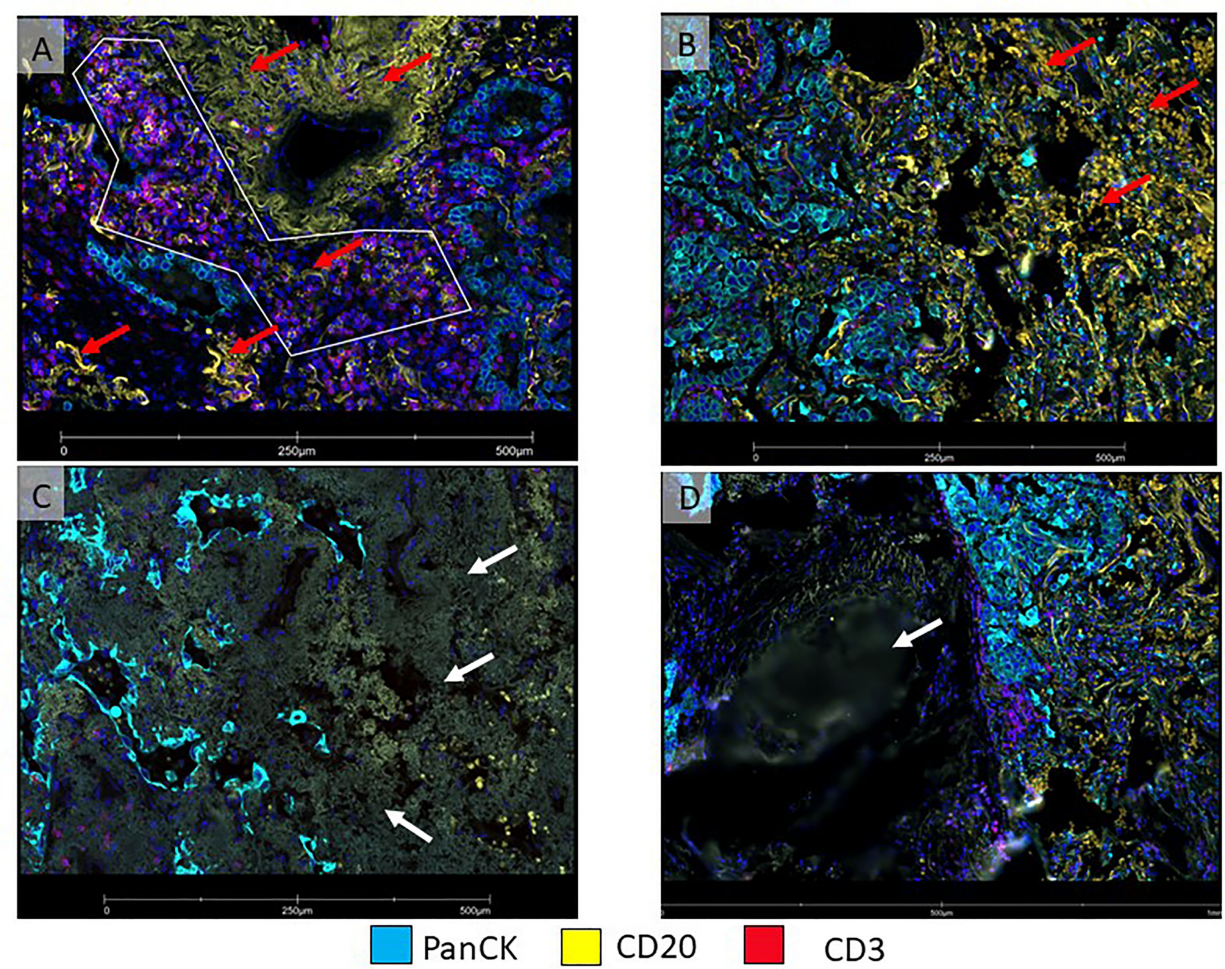
Micro photographs showing artifacts in immunofluorescence DSP slide from a non-small cell lung carcinoma tumor sample **(A)** ROI was drawn with a polygon ROI, avoiding elastic fibers (red arrows) that emit non-specific fluorescence signal (yellow), **(B)** Area on the right (red arrows) show numerous red blood cells emitting non-specific fluorescence signal (yellow). Both, elastic fibers and red blood cells, interfered with segmentation for B-cells. **(C)** Tumor area on the left shows fibrosis with non-specific fluorescent signals (white arrow). **(D)** An area out of focus (white arrow) is observed on the left side of the image.

Different types of ROIs can be selected depending on the aims of the study and the tissue characteristics. These ROIs can be geometric ROIs with standard shapes (rectangles, squares, and circles) that are easy to manage and reproduce; geometric polygons that can be used to assess tissue heterogeneity are the preferred method when segmentation of different compartments is not possible due to limitations of biomarker expression patterns (**Figure 3**). It is also the best option to avoid artifacts that may produce background fluorescence, such as red blood cells and elastic fibers (**Figure 4**). Contour profiling is another option where segmentation is performed with concentric and non-concentric parallel bands and is used to evaluate the oligo expression from a central structure using radiating ROIs. Whole tissue gridding uses geometric ROIs that can be placed at regularly defined intervals, right next to the other, until the entire sample is profiled. Of note, a single ROI size can be, at a minimum, 5 μm × 5 μm and, at most, a rectangle of 680 μm × 785 μm (18).

### ROI Segment Profiling

**Figure 5:**
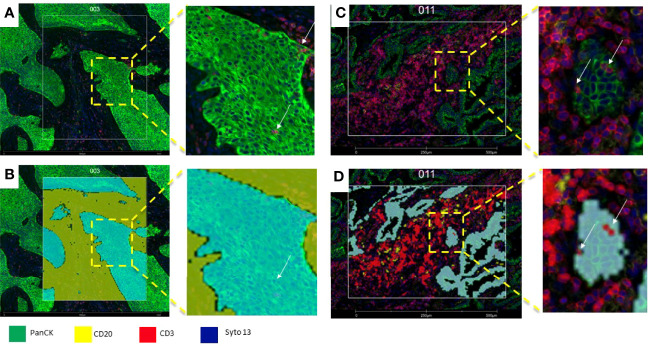
Microphotograph of immunofluorescence sections of surgical resected non-small cell carcinoma **(A)** Squamous cell carcinoma; **(B)** adenocarcinoma) using DSP assay with PanCK (tumor), CD3 (T-cells), CD20 (B-cells) and Syto13 (nuclei) as visualization markers. **(B, D)** illustrates different segmentation strategies for tumor, stroma and T cells. In B the segmentation was performed in tumor (cyan mark up) and stroma (yellow mark up) segments based in panCK expression, with this strategy, tumor segments include tumor cells and Intra-epithelial immune cells (white arrows), and stroma segments include all tissue elements among tumor segments **(B)**, In **(C)**, segmentation was performed in tumor-(cyan mark up), B-cell (yellow mark up) and T-cells (red mark up) segments based in cell biomarker profile, intra-tumor T-cells (white arrows) are included in the T-cell compartment **(D)**.

After placing the ROIs, we can further divide them into different biological compartments based on the morphology markers’ expression. A segment profiling refers to phenotypic or distinct biological architectural compartments revealed by the morphology markers, with the most common compartments being “Tumor” and “Stroma” (tumor marker +) vs. “Stroma” (tumor marker -) (84), but we can also perform cell type-specific profiling for distinct cell populations with cell type-specific biomarkers, such as CD45+, CD3+, or CD68+ (**Figure 5**).

### Adjusting the Software Parameters for ROI Segmentation

The image analysis software of the DSP device has options for the adjustment of the parameters to recognize different compartments identified by morphology biomarkers. These parameters may change due to histological variability among samples and among ROIs from the same sample. Harmonization of ROI is highly encouraged, and the definition of specific compartments must be clearly stated before starting any project (e.g., Tumor vs. Immune, Tumor vs. Stroma). The parameters used to modify the ROI segmentation are as follows: “segment definition”, which determines the cellular composition of each AOI (tumor, stroma, and immune); “erosion”, which effectively increases the boundary between segments; “N-dilation”, which enlarges the UV-light mask in an AOI around the detected nuclei; “Hole size”, which fills holes in the AOI that are smaller than the value set; and “particle size”, which eliminates any AOI particles smaller than the value (2 µm) (48) .

### Setting the Collection Order

Another important parameter is the collection order of the different segments set by the morphology biomarkers, as the order in which they are selected on the software will determine the order in which UV light illuminates each AOI within an ROI. It is recommended to collect AOIs from low to high abundance. Of note, to avoid acellular materials such as elastic fibers that co-express multiple morphology markers (**Figure 4**), an additional segment in the GeoMx platform that excludes co-expressed parameters from analysis can be included.

### Pathology Quality Control After Assay Completion

Once the processes described above are completed, protein or RNA counts will be obtained. To procure high-quality data, quality control of the entire set and ROIs selected is performed in two steps.

**Figure 6:**
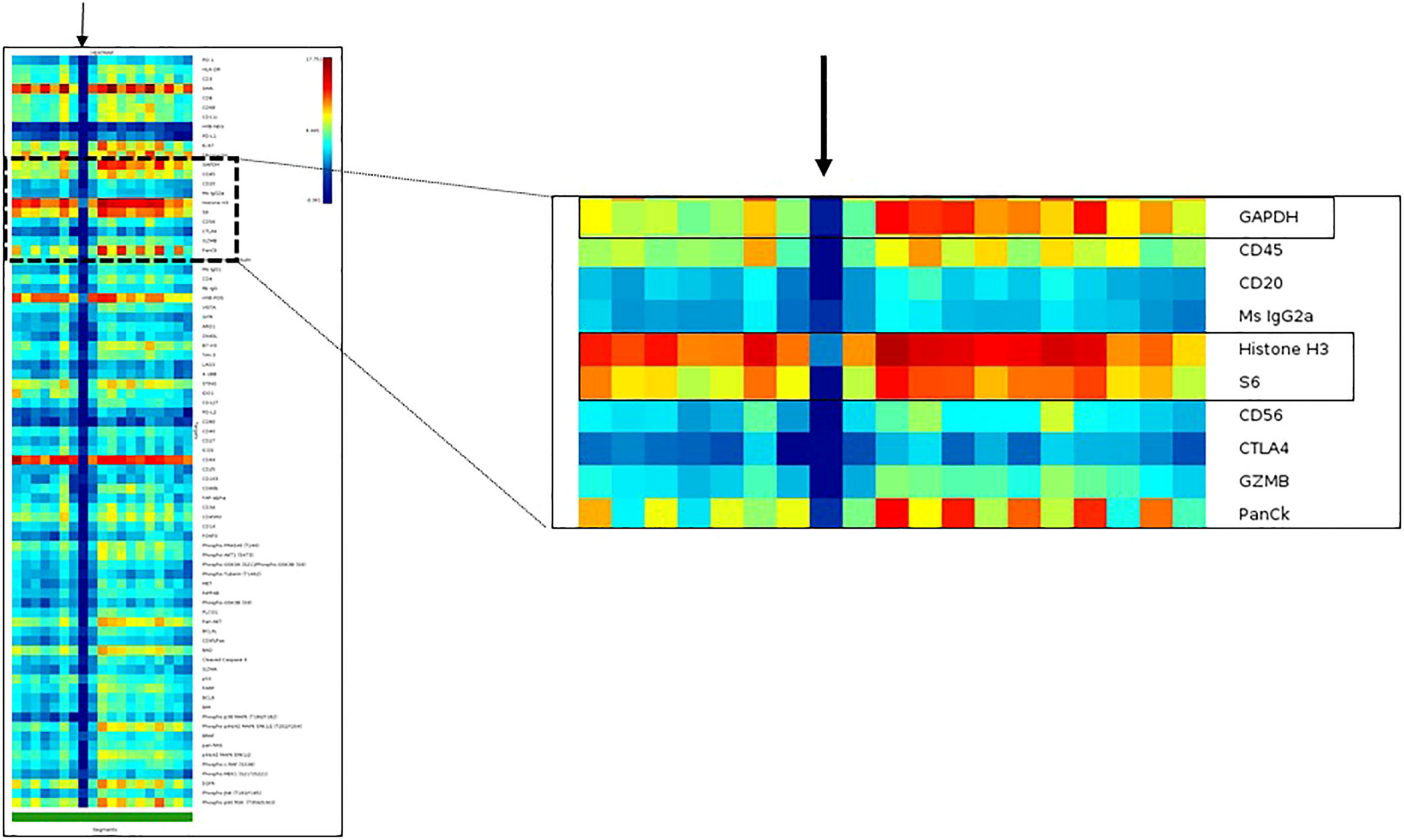
Heatmap of an initial dataset obtained with DSP assay to illustrate the visualization of data as quality control tool. The DSP counts of one region of interest indicated with a black arrow show no or very low DSP counts from all targets of the DSP protein panel, including the housekeeper proteins: GAPDH, Histone3 and S6. The DSP quality control report showed a positive control normalization tag in this specific region of interest.

*a) Assay QC with the initial dataset:* After a study group is created, the first step is to evaluate the initial dataset. This can be easily accomplished through an initial heatmap in the GeoMx Data analysis suite. With this tool, the researcher can assess the protein expression of each segment, first comparing the expression of the housekeepers (which should be higher and present in all the AOIs) and then comparing the relationship between the observed and expected compartment-specific expression of proteins (PanCK in tumor areas of carcinomas and CD45 in immune areas). Furthermore, the researcher can easily notice AOIs that lack adequate expression of markers by the presence of a blue vertical line on the heatmap, which translates to low expression of all the markers. Such an expression pattern is commonly seen with evaporation issues during the collection step of the assay (**Figure 6**).
*b) Platform QC:* After the initial QC, the user must run the software’s QC with the initial dataset, during which the software examines each AOI and gives a warning sign if a parameter below the optimal quality limits is detected. The parameters included in this QC include FOV registration and binding density, positive control normalization, and minimal nuclei and surface area. AOIs with warnings should be considered for exclusion for the analysis after a careful review by the pathologist, comparing the causes of the warnings with protein expression of compartments. As an example, the image analysis software counting nuclei algorithm may not perform optimally for all samples due to tissue heterogeneity, and ROIs with a low nuclei count warning must be visualized to confirm whether exclusion from analysis is appropriate.
c) *Biomarker expression quality control:* Even if the AOIs pass the initial evaluation and platform QC, we test the accuracy of some biomarkers included in the DSP panel using the count data obtained and by comparing areas that should express different biomarkers in different biological compartments. For example, epithelial tumor segments should have higher counts of CK compared to immune cell segments. Similarly, immune cell segments should have higher counts of CD45 or other immune-related biomarkers such as CD3, in the “immune segments” compared to “tumor segments”. Of note, some carcinomas may biologically express lower levels of CK; however, this feature should be noted with a morphology biomarker (CK) when performing ROI selection.
*d) Custom Scripts:* The GeoMx Data analysis suite allows the user to perform nanoString and custom R-scripts to evaluate different normalization strategies by correlating biomarker expression among samples and among AOIs (85). The nanoString scripts are helpful as they offer a broad visualization of the behavior of the samples, being able to detect batch effects or outliers. The scripts also provide information about the expression of the biomarkers compared to the background to evaluate expected biomarker expression across ROIs and to correlate housekeepers and IgG markers.

### Data Normalization

Data normalization must be performed based on certain features of samples, strategies for ROI selection and segmentation, and the expression of housekeepers or isotype biomarkers. For this task, the platform has different options:

a) *Scale to nuclei:* This method is better used when studying biomarker expression per cell. The ratio of the geometric mean of nuclei to the measured number of nuclei is used to adjust the counts in the segment. Currently, we do not recommend this method since the algorithm for detection of nuclei count is not accurate due to diverse histological features usually found in tissue samples.
b) *Scale to area:* This normalization is applied when the size of ROIs differs significantly in the same sample or between samples. The software calculates the geometric mean of the areas and adjusts the counts detected in the segments.
c) *Housekeepers (reference normalization, RN):* This method uses endogenous reference targets to adjust for differences in protein abundance or quality in the sample. There are four reference transcripts (UBB, OAZ1, SDHA, and POLR2A) in RNA panels and three antibodies against cellular proteins (GAPDH, Histone H3, and S6) in protein panels. Before choosing a Housekeeper normalization method, it is important to QC the potential normalization factors, which is done by evaluating their signal strength correlation. After we select the factors with better correlation, we choose their geometric mean for calculations.
d) *Background correction (SNR):* Background correction allows the user to adjust any non-specific target adherence to the tissue. There are eight negative probes for RNA panels, and three isotype IgGs included in the protein panels (mouse IgG1, IgG2a, and rabbit IgG). In SNR, it is also important to evaluate the correlation of the negative probes or isotypes before starting the normalization in order to choose those with better behavior (better correlation) across the tissue. Protein or RNA profiles of single or few cells can be difficult to define the above background. Markers with low counts should be looked at with caution (71) (e.g., PD-L1), especially since counts below 1 (found, for example, in immunologically cold areas) are equalized to 1 in the initial dataset, which can alter the final data when normalized by SNR (86). It has been previously recommended to normalize to ERCC, scale counts to nuclei counts, and then normalize to HK proteins or IgG controls (15). The methodology to select the normalization strategy is still not standardized. It is recommended to work closely with a bioinformatician, a data analyst, or a statistician to test different normalization approaches so that scenarios in which DSP counts are artificially increased or decreased after normalization may be avoided. These approaches will be defined by the initial dataset analysis including area size, nuclei count, and expression of housekeeping genes and isotype controls across all tumor samples. We have observed that AOIs of similar area are best for analysis, but tissue heterogeneity may not allow this, especially when the segmentation approach is used.

### Data Analysis on the GeoMx Platform

Once the data pass QC and are normalized, the user can upload relevant annotated information to the software using the “Manage Annotation” tool. We use the visualization tools of the GeoMx Data analysis suite to have a general overview of the protein or RNA counts. The heatmap, the first visualization tool we use, permits the construction of customized unsupervised clusters by selecting specific AOIs and biomarkers. For statistical tests, the platform gives many options such as unpaired *t*-tests (useful for comparing two groups of independent samples), paired *t*-tests (useful for comparing two groups with a natural paired structure), Mann–Whitney *U*-test (useful for data that are extremely skewed or heavy-tailed), and linear mixed models (useful for data with repeated measurements from each sampling unit, e.g., multiple ROIs from each sample) (71). These results can be visualized in different ways such as volcano plots. The hypothesis and aims of each project will help us to decide which statistical test is better suited for our purposes.

## Comparison of DSP and Other Multiplex and High-Plex Tissue-Based Platforms

**Spatial Gene Expression (Visium):** This is a probe-based spatially resolved transcriptomic platform that profiles frozen or FFPE tissue. Compared to the DSP platform, Visium has higher resolution; it can provide information of 1 to 10 cell resolutions on average per spot in an entire section, and each capture spot is limited to a diameter of 55 µm, with a 45-µm gap between spots (87) (88). The tissue size is limited to 6.5 × 6.5 mm per capture area, which implies that the tissue processing requires special handling, and large-sized tissues need to be sectioned in different fragments that can fit into the capture area; thus, morphology assessment of a preview H&E slide is needed to design sectioning. Furthermore, before the Visium assay, RNA quality must be assessed by sectioning curls of tissue to determine RNA integrity (89). This extra handling of the tissue is not ideal for small samples or to identify lesions that are small and may be lost in subsequent sections. As an alternative, the Visium platform offers a device (CytAssist) that can transfer transcriptomics and proteomic analytes from the standard glass slides onto the Visium slides, allowing a preview of the tissue samples (90).

Spatial information on well-annotated specific areas guided by protein expression (CD3+ for T cells and PanCK+ for tumor cells) or morphology features can be potentially performed using immunofluorescence or H&E staining. However, this assay is only limited to gene expression and does not include high-throughput protein assessment.

- **Multiplex Immunofluorescence (mIF):** This technique uses mIF panels with a tyramide amplification system for the evaluation of expression of proteins of interest, which correspond to specific cell types and biological processes. In contrast with the DSP, mIF provides single-cell density and *x* and *y* cell coordinates, which, in turn, provide original spatial arrangement of the cells in the tissue, and facilitates the study of specific phenotypes and its biological interactions with tumoral morphological characteristics (91). Because of the spectrum of fluorophores used, mIF is limited to 8 antibodies per panel; however, distinct panels can be tailored to individual projects (27). mIF requires digital image analysis supervised by a pathologist with longer processing times than DSP. Similar to DSP, whole tissue section analysis can be performed using “grid” tissue analysis tools (92); since throughput is limited by the time spent performing digital image analysis of mIF images, ROI selection is preferred.
- ***In Situ* Hybridization Techniques for RNA and Other Target Sequences:** *In situ* hybridization assay allows the detection of a target nucleotide sequence (e.g., DNA, RNA, and miRNA) in tissue, allowing the *in situ* visualization of the target sequence within intact cells. One of the most common and commercially available options compatible with FFPE uses ACD’s patented probe design to amplify the target-specific signals and visualize them using chromogens or fluorescent dyes, which can also be combined with IHC and can be used as VM in the DSP platform (93–95). This technique has the advantage of interrogating RNA, miRNA, or other targets that are present in the TME, validating differentially expressed genes identified by other techniques that quantify bulk RNA or miRNA from tissue (96, 97), and evaluating other targets that are not included in the RNA DSP panels such as CAR-T sequences (98). This platform provides a more accurate information on the presence and absence of the expression of the targets, while the DSP does not provide accurate information on the absence of expression of specific targets since normalization strategies can potentially amplify noise.
- **Spatial Phenotyping (PhenoCycler):** Formerly known as CODEX platform, this platform uses oligonucleotide-conjugated antibodies and sequential fluorescent reporters to detect up to 60 markers simultaneously in a single FFPE tissue section at resolutions that resolve individual cells, generating information on the distribution of different cellular phenotypes and their morphological spatial context (99, 100). Although the DSP has a higher number of analytes, the PhenoCycler provides single-cell resolution information and phenotyping in whole tissue sections, with the possibility of designing and validating special biomarker panels (101). In addition to its protein biomarker assays, the platform will soon be enabled with RNA detection using the new PhenoCycler-Fusion system (102), which could be compared in the future to a new nanoString technology called “CosMx” that integrates protein analysis with RNA and also offers the possibility of single-cell resolution (103, 104). From a histotechnical point of view, one of the disadvantages of the PhenoCycler compared to the DSP is the challenge to process tissue section and slide preparation, because this assay uses a coverslip of a limited size (22 × 22 mm) (105).
- **Multiplexed Ion Beam Imaging (MIBI):** MIBI is performed by staining tissue with a panel of antibodies tagged with monoisotopic metal reporters and then imaging the tissue using secondary-ion mass spectrometry (106, 107). MIBI allows the simultaneous detection of 40+ markers at subcellular resolution in FFPE or frozen tissue, enabling single-cell segmentation, cell type classification, and spatial analysis of the cells present in the TME. In this platform, non-specific binding between antibodies and epitopes can make the validation and standardization of biomarkers a challenge and proper controls are needed for staining and imaging (108).
- **Imaging Mass Cytometry (IMC):** IMC is a technology that combines laser ablation and cytometry by time of flight for the detection of targets labeled with metal-tagged antibodies in frozen tissue or FFPE sections. Compared to the DSP, IMC offers the analysis of up to 40 markers on a tissue section at a single-cell level, and similarly, it uses an ROI selection strategy with the possibility of compartment segmentation based on biomarker expression. Also, IMC can give a resolution of 1,000 nm in the first scan, and one area can be re-scanned to obtain a resolution as low as 260 nm. However, the use of this technology requires the thorough design and validation of complex panels adapted to various tissues and diseases, and the tissue ablation process means that the sample cannot be re-used after the analysis in this platform (109, 110).
- **Multiplexed Error-Robust Fluorescent *In Situ* Hybridization (MERFISH):** MERFISH is a single-cell transcriptome-scale RNA imaging method that uses error-robust barcodes to measure RNA transcripts in tissues. This is achieved by physically imprinting the barcodes on RNAs, and then measuring these barcodes through sequential rounds of imaging (111, 112). Compared to the DSP, MERFISH offers single-cell and subcellular resolution in a whole tissue section. Also, the platform works mostly with frozen tissue sections but is currently validating an FFPE workflow (113).

## Conclusions

Recent advances in tissue-based biomarker development assays have shown that high-plex immunoprofiling is feasible and useful in translational immune-oncology research. In this review, we described the DSP platform from a technical point of view, plus current evidence of the advantages and limitations of the applications of this technology in translational immune-oncology research and in the clinical setting.

The capabilities of the platform in cancer research can be generalized to a variety of tumors with high heterogeneity of cell types. Although the commercially available morphology biomarkers are limited (25, 26, 44), the platform has the flexibility to use different morphology biomarkers that can be customized by the laboratory and be tailored to specific questions. Of note, morphology immunofluorescence biomarkers can be analyzed using digital image analysis. Images can be exported and used with different image analysis software that can provide spatial information of x and y coordinates of different cell phenotypes, but they are limited to only 3 tumor and/or immune markers plus the nuclear marker, giving limited results when compared to high-plex immunofluorescence technologies.

It is worth mentioning that DSP panels have also been designed for non-neoplastic diseases such as COVID-19 (COVID-19 Immune Response Atlas) (114) or the study of neurologic diseases such as Alzheimer’s disease (115). These assays use a similar approach to what is used in solid tumors, with morphology markers to identify spatial areas of biological interest related to the diseases, and can be potentially used in oncology research.

Several aspects of this platform could be improved. Information obtained from pathology-guided and well-annotated ROIs could be used in computational pathology and artificial intelligence algorithms to better understand tumor biology and eventually guide biomarker-driven clinical trials. Also, H&E image availability in the platform and integration with immunofluorescence DSP images would be of great interest for researchers. Ongoing efforts to fully automate the technical aspects of tissue preparation would potentially help to improve the many reported limitations related to technical variability of assays among different users. Single-cell resolution and the simultaneous assessment of protein and RNA in the same slide would also have an impact on the capabilities of this high-plex platform.

## Author Contributions

SH and RL contributed to the review concept and the drafting and writing of the manuscript. LS contributed to the review concept, outline, drafting, writing, and editing of the manuscript. All authors listed have made a substantial, direct, and intellectual contribution to the work and approved it for publication.

## Funding

This project was supported in part by the Translational Molecular Pathology Immunoprofiling Laboratory (TMP-IL) Moonshot platform at the Department Translational Molecular Pathology at the University of Texas MD Anderson Cancer Center. Scientific and financial support for the CIMAC-CIDC Network is provided through the National Cancer Institute (NCI) Cooperative Agreements U24CA224285 (to the MD Anderson Cancer Center CIMAC).

## Conflict of Interest

The authors declare that the research was conducted in the absence of any commercial or financial relationships that could be construed as a potential conflict of interest.

## Publisher’s Note

All claims expressed in this article are solely those of the authors and do not necessarily represent those of their affiliated organizations, or those of the publisher, the editors and the reviewers. Any product that may be evaluated in this article, or claim that may be made by its manufacturer, is not guaranteed or endorsed by the publisher.
